# Use of prenatal care and hospital structure according to obstetric risk in Rio de Janeiro, Brazil

**DOI:** 10.11606/s1518-8787.2025059006571

**Published:** 2025-10-20

**Authors:** Sonia Duarte de Azevedo Bittencourt, Rosa Maria Soares Madeira Domingues, Danielle Portella Ferreira, Ana Paula Esteves-Pereira, Marcos Augusto Bastos Dias, Marcos Nakamura-Pereira, Alessandra do Nascimento Bernardo, Paulo Cesar da Graça Souza Suppo Blengini, Maria Auxiliadora de Souza Mendes Gomes, Maria do Carmo Leal

**Affiliations:** I Fundação Oswaldo Cruz. Escola Nacional de Saúde Pública Sérgio Arouca. Rio de Janeiro, RJ, Brasil; II Fundação Oswaldo Cruz. Instituto Nacional de Infectologia Evandro Chagas. Rio de Janeiro, RJ, Brasil; III Fundação Oswaldo Cruz. Instituto Nacional de Saúde da Mulher, Criança e Adolescente Fernandes Figueira. Rio de Janeiro, RJ, Brasil

**Keywords:** Prenatal Care, Structure of Services, Quality of Health Care

## Abstract

**OBJECTIVE:**

To estimate the use and adequacy of prenatal care services and to verify the structure of the maternity hospital to meet the needs of health care during childbirth in the Brazilian Unified Health System.

**METHODS:**

This is a cross-sectional, hospital-based study conducted in the state of Rio de Janeiro, Brazil. 1,073 puerperae were eligible, whose delivery took place in public and mixed hospitals. The interviews were conducted in the hospital, and data were extracted from the prenatal card and maternal medical records. To evaluate the hospital structure, managers were interviewed. Sociodemographic, obstetric and prenatal care characteristics of the puerperae were described according to obstetric risk. The evaluation of the structure of the maternity hospital included: human resources, medicines, emergency equipment, and support services, according to the complexity level. Moreover, the distribution of puerperae classified as at obstetric risk was analyzed according to the complexity of the hospital structure.

**RESULTS:**

The highest prevalence of high obstetric risk was observed in hospitals located in small cities, among white women, aged 35 years or over, with up to 11 years of formal study, multiparous, and obese. Prenatal care adequacy, control of hypertension, diabetes, and nutritional evaluation were low, and traveling for delivery was significant. Hospitals with ICU/NICU presented better levels of adequacy in all structure dimensions evaluated. At the state level, 30.5% of women at high obstetric risk were seen in maternity hospitals without ICU/NICU; and 40.4% women at normal risk, in maternity hospitals with ICU/NICU.

**CONCLUSIONS:**

We highlight the need to improve prenatal care and the implementation of an articulated network of services that integrate the Brazilian Unified Health System (SUS), centered on maternity hospitals that guarantee the structure conditions for quality and safety in obstetric and neonatal care, both in good practices at normal risk and in the management of complications at high obstetric risk, in such a way to impact the considerable outcomes of maternal and neonatal morbidity and mortality.

## INTRODUCTION

In Brazil, although prenatal and labor care have practically reached universal coverage, with almost all deliveries occurring in hospitals and assisted by qualified personnel^1,2^, the persistence of high levels of maternal morbidity and mortality due to preventable causes^3,4^ indicates problems associated with work processes during obstetric care and the management of services of the care networks for sexual and reproductive health^5,6^. These are situations that contribute to increasing the inequities of pregnant women in using healthcare services appropriate to their needs as well as in the outcomes of the provided care.

Aiming at changing this scenario, the Brazilian Ministry of Health implemented, in 2011, the *Rede Cegonha* [Stork Network] strategy^7^, which is founded on the development of the line of care for pregnant women based on the classification of each woman's health needs, in order to structure the articulation of the responsibilities and functions of each healthcare service involved in prenatal and labor care, thus ensuring comprehensive, timely, and problem-solving care.

However, more than a decade after the implementation of Rede Cegonha, even when risks are identified during the prenatal period, it is difficult to guarantee hospitalization in maternity hospitals with a compatible structure, considering the care needs of both the mother and the newborn^8,9^.

This situation becomes more difficult in the obstetric emergency, which demands the agility of the reference mechanisms. The difficulty of communication between the services and the control centers responsible for enabling the transfer to services with a compatible structure has been pointed out as a factor associated with the delay in receiving adequate care^3^. The regulation of high maternal risk care, one of the challenges to be overcome for the organization of maternal and child care, is part of the priorities of the State Health Plan (2020–2023)^10^ of the state of Rio de Janeiro (RJ), Brazil.

In this study, our objective was to estimate the use and adequacy of prenatal care services and to verify the structure of maternity hospitals to meet the healthcare needs during childbirth in the Brazilian Unified Health System (SUS) in RJ.

## METHODS

This is a cross-sectional, hospital-based, and of national coverage study ("Nascer no Brasil II" [Birth in Brazil II] — NBII). A two-stage probabilistic sample (hospital and puerperae) was selected, according to the study protocol published in Leal et al.^11^. For this study, puerperae of live births (LB) with any weight or gestational age and puerperae of stillbirths with gestational age ≥ 22 weeks or ≥ 500g weight were eligible.

### Study in Rio de Janeiro

In RJ, the puerperae sample was estimated based on the proportion of cesarean sections (57%) in 2019, a 5% significance level, and 90% power to detect differences of 7%. A design effect of 1.3 was used, resulting in the minimum sample size of 1,350 puerperae. To reach this number, the sample of puerperae from RJ was 30 in facilities with 100–499 LB/year and 90 in hospitals with ≥ 500 LB/year. *Post-hoc* calculations indicate that the sample has the power to detect absolute differences of 5% for outcomes with prevalence between 5 and 20%.

A total of 17 hospitals classified as public (source of funding and management by the public sector) and mixed (public and private sources of funding and private management) were included in this analysis, representing 86 hospitals in the RJ. In public hospitals, all puerperae interviewed were included, while in mixed hospitals only those who had a public source of funding for delivery were included, corresponding to 86.5% of those interviewed in these hospitals.

Data collection, carried out from 2021 to 2023, included interviews with women in the immediate puerperium, data extraction from prenatal cards, and clinical data from maternal medical records after hospital discharge. Puerperae who did not present data from prenatal cards were excluded from the analysis. Information on maternity hospitals’ structure was obtained from an interview with the manager of each hospital unit. Detailed information on the collection instruments is reported in Leal et al.^11^.

In order to estimate the healthcare needs in each of the maternity hospitals of the study, the puerperae were classified according to obstetric risk^8^. Those who presented at least one of the following clinical and/or obstetric conditions identified in prenatal care were considered high-risk: chronic or gestational hypertension, pregestational or gestational diabetes, chronic kidney disease, heart disease, lupus, scleroderma, HIV infection, other infections upon hospital admission, and twin pregnancy. The other puerperae were classified as normal risk. The following variables were analyzed by obstetric risk: hospital location (metropolitan region and small cities), hospital type (public and mixed), age (10–19, 20–34, and 35 years or over), skin color (white and Black), years of formal study (up to 11, 12 or over), parity (primiparous and multiparous), and pregestational body mass index (BMI < 25, BMI ≥ 25).

Prenatal care was verified according to obstetric risk by means of the following indicators: prenatal care adequacy (onset up to 12 weeks of pregnancy and number of appointments according to gestational age, as per the schedule recommended by the World Health Organization^12^); type of health facility where the woman had the prenatal appointments; woman's body weight record on three occasions; height record; reason for blood pressure measurement per appointment; and blood glucose tests. The integration between prenatal care and delivery care was analyzed by the proportion of women who received guidance on the place of hospitalization for delivery, the proportion of women who sought more than one service for hospitalization for delivery, and the reasons for traveling for delivery.

To estimate the level of complexity of delivery care, the sample hospitals were classified according to the existence of Adult Intensive Care Unit (ICU) and Neonatal Intensive Care Unit (NICU). For high-complexity maternity hospitals, women were inquired whether it was a formal reference by the regulation system for delivery of pregnant women at risk. To investigate the "human resources" dimension, the following were investigated: availability of at least one of each professional (obstetrician, neonatologist, anesthesiologist, obstetric and neonatal nurse) on a 24-hour on-call shift; the existence of medical and nursing coordinator specialist in obstetrics and neonatology; the availability of backup specialists for support in the case of women with complications; and whether the hospital was a teaching facility for medical and/or nursing residency in obstetrics and/or neonatology. In the "medicines" and "equipment" dimensions, the availability of 20 drug classes^13^ and a set of equipment for emergency care to pregnant women and newborns was verified^13^. Regarding the "support services" dimension, access to a blood bank or transfusion unit, laboratory of pathologies and clinical analysis, imaging tests, and ambulance for the transportation of the mother and the newborn was verified.

Subsequently, the distribution of puerperae classified as at obstetric risk was analyzed according to the complexity of the hospital structure by hospital type and location. For the comparison between the categories, the χ^2^ test was used, considering p < 0.05 as significant.

All analyses were carried out using the Statistical Package for the Social Sciences (SPSS) software, version 21 (SPSS Inc., Chicago, United States), with the inclusion of the design effect, weighting, and calibration. For the RJ sample, groups composed of the combination of stratum, type of delivery (vaginal, cesarean section), and women's age (10–19, 20–34, ≥ 35) were used for calibration, using SINASC-2022 (Live Birth Information System [*Sistema de Informação sobre Nascidos Vivos* – SINASC] as reference.

The NBII research was approved by the National Commission of Ethics in Research, Certificate of Presentation for Ethical Consideration (CAAE): 21633519.5.0000.5240, on March 11, 2020, and, in its absence, it was approved by the local research ethics committees of the institutions or the clinical board's

## RESULTS

We analyzed 1,073 puerperae, corresponding to 82.2% of the sample of women interviewed in the maternity hospitals with public source of funding. Of the total women analyzed, 266 (24.8%) were classified as at high obstetric risk. We observed a higher prevalence of high obstetric risk in hospitals located in small cities of the state, among white women, those aged 35 years or over, with up to 11 years of formal study, multiparous and obese women. Prior to hospitalization for delivery, 18.6% of women at high obstetric risk reported to have been hospitalized during pregnancy, a percentage almost three times higher than that found among women at normal risk (Table 1).

**Table 1:** Number and percentage of women at high obstetric risk according to hospital and maternal characteristics. State of Rio de Janeiro, 2021–2023.

		Obstetric risk		Total n	%	p-value^a^
		Low	High			
Total		807	266	1,073	24.8	
Location						
	Metropolitan Region	599	169	768	22.0	0.00
	Small cities	208	97	305	31.8	
Hospital type						
	Public	694	211	905	23.3	0.00
	Mixed	113	55	168	32.7	
Age group (years)						
	10 to 19	119	27	146	18.5	0.00
	20 to 34	605	188	793	23.7	
	35 or over	84	51	135	37.8	
Skin color						
	White	178	78	256	30.5	0.05
	Black (Black, mixed-race and Indigenous)	629	188	817	23.0	
Years of formal study						
	Up to 11	422	163	585	27.9	0.01
	≥ 12	385	103	488	21.1	
Parity						
	Primiparous	350	94	444	21.2	0.01
	Multiparous	457	173	630	27.5	
Body Mass Index						
	Up to 24	528	138	666	20.7	0.00
	≥ 25	154	88	242	36.4	
Was the puerpera hospitalized during this pregnancy?						
	No	744	214	958	6.7	0.00
	Yes	54	49	103	18.6	

a p-value of the χ^2^ test.

As observed in Table 2, the prenatal care adequacy, considering the time of onset and number of appointments, was 71.5%, being a little more than 90% of pregnant women seen in a single health facility, preferably in primary health care. Blood pressure measurement was recorded in 88.1% of the appointments, first blood glucose in 78.1%, and second blood glucose in 30%. Only one third of the pregnant women had a complete record of blood pressure and blood glucose tests. The weight was recorded at three moments of prenatal care in 90% of prenatal care cards, but the height record was incomplete, limiting nutritional assessment (40% in women at high obstetric risk). Women at high obstetric risk had the second blood glucose test recorded the most, with no other differences in relation to those at normal risk. Only 16.8% were assisted in outpatient reference units.

**Table 2:** Number and distribution of prenatal care indicators of puerperae according to obstetric risk, Rio de Janeiro, 2021–2023.

		Obstetric risk		Total n	Obstetric risk		Total %	p-value^a^
		Low n	High n		Low %	High %		
Adequacy of the number of appointments according to gestational age at delivery								
	Yes	566	194	760	70.8	73.4	71.5	0.40
	No	233	70	303	29.2	26.5	28.5	-
Organization of prenatal care								
Prenatal care received only in one type of health facility								
	Yes	747	239	986	92.5	89.8	91.8	0.33
	No	60	27	87	7.5	10.2	8.2	-
Type of health facility where the woman had prenatal appointments								
	Primary Health Care	623	179	802	83.5	74.9	81.4	0.00
	Outpatient clinic (public, private and health insurance)	65	40	105	8.7	16.7	10.7	-
	Others (group consultation, birth center)	58	20	78	7.8	8.4	7.9	-
Procedures								
	Weighing at 1st appointment, 24th to 28th weeks, and last appointment	651	219	870	85.4	88.7	86.2	0.53
	Height measurement	405	110	515	50.2	41.4	48.0	0.01
	All measurements of weight and height	355	100	455	44.0	37.6	42.4	0.06
	Less than one blood pressure measurement per appointment	668	221	889	87.8	89.1	88.1	0.57
	1st blood glucose test	634	204	838	78.6	76.7	78.1	0.52
	2nd blood glucose test	255	112	367	31.6	42.1	34.2	0.02
	Blood pressure (at least one per appointment) and blood glucose tests	229	96	325	28.4	36.1	30.3	0.01
Received guidance to visit the maternity hospital		121	30	151	15.0	11.3	14.1	0.12
Traveling for delivery		121	48	169	15.0	18.0	15.8	0.08
Reasons for traveling								
	There was no vacancy	21	8	29	17.8	17.8	17.8	0.72
	The woman did not go into labor	37	14	51	31.4	31.1	31.3	0.65
	Referred to another maternity hospital due to risk status	18	14	32	15.3	31.1	19.6	0.01
	Referred to another maternity hospital, but it was not due to risk status	18	2	20	15.3	4.4	12.3	0.12
	Maternity hospital without doctor or conditions to provide care	6	5	11	5.1	11.1	6.7	0.11
	Other	18	2	20	15.3	4.4	12.3	0.12

a p-value of the χ^2^ test.

Still in Table 2, only 14.1% of the pregnant women were instructed to visit the maternity hospital to know the environment in which they would stay during labor and delivery, and the difference by obstetric risk was nonsignificant. We verified that traveling for delivery was more frequent among women at high obstetric risk, although also without statistical significance. The main reasons mentioned were similar between the two groups: there was no vacancy and the woman was not in labor, except for cases where the reason for being referred to another maternity hospital was a risk status, reported by over a third of women at high obstetric risk.

In Table 3, we show that 30.2% of the hospitals in the sample had ICU/NICU, being 34.3% public and 15.8% mixed hospitals, with no difference in the distribution between the metropolitan region and small cities. Of these maternity hospitals, 46.1% reported being a formal reference by the regulation system for hospitalization and delivery of pregnant women and newborns at risk (data not presented in the tables).

**Table 3:** Distribution of hospitals by location, type of source of funding, and availability of human resources according to level of complexity, Rio de Janeiro, 2021–2023.

		ICU and NICU				p-value^a^
		No		Yes		
		n	%	n	%	
Hospital type						
	Public	44	65.7	23	34.3	0.12
	Mixed	16	84.2	3	15.8	-
Location						
	Metropolitan Region	40	71.4	16	28.6	0.64
	Small cities	20	66.7	10	33.3	-
Human resources Obstetrics (24-hour on-call shift)						
	Obstetrician	60	100	26	100	-
	Anesthesiologist	48	80.0	26	100	0.01
	Obstetric nurse	40	66.7	19	73.1	-
	Total	16	26.7	19	73.1	0.00
	Medical coordinator specialist in obstetrics	60	100	26	100	-
	Nursing coordinator specialist in obstetrics	17	28.3	21	80.8	0.00
Neonatology (24-hour on-call shift)						
	Neonatologist	2	3.3	26	100	0.00
	Anesthesiologist	3	5.0	5	19.2	0.03
	Neonatal nurse	12	20.0	19	70.4	0.00
	Total	0	0	5	18.5	
	Medical coordinator specialist in neonatology	18	69.2	26	100	0.00
	Nursing coordinator specialist in neonatology	6	23.1	26	100	0.00
Presence of specialists^b^						
	None	24	40.7	0	0	0.00
	Total	3	5.1	10	37.0	0.00
Offer of medical and/or nursing residency in obstetrics or neonatology		4	6.7	17	63.0	0.00

ICU: adult intensive care unit; NICU: neonatal intensive care unit.

a p-value of the χ^2^ test.

b Medical specialty: Cardiology, Neurology; Nephrology; Hematology; Infectious diseases; and General Surgeon.

Lower proportions of human resources available on a 24-hour on-call shift were reported in low-complexity hospitals, mainly neonatologists (3.3%), anesthesiologists (5%), and nurses (20%) in the neonatology service. The availability of anesthesiologists was infrequent even in high-risk services (19.2%), differing from the other professional categories, in which this availability was higher than 70%. Low-complexity hospitals present the lowest frequency of medical and nursing coordinators in obstetrics and neonatology services, and of backup specialist doctors for cases of women with complications (40.7%). Only 6.7% of hospitals offer medical and/or nursing residency programs (Table 3).

In Table 4, we show the availability of essential and strategic equipment for maternal and neonatal care in emergencies. For women's care, the non-pneumatic anti-shock garment (NASG) was the least frequent equipment for all maternity hospitals, whereas the intrauterine balloon was not reported in any low-complexity maternity hospital and in less than half among those of high complexity. For newborn's care, with the exception of the low availability of baby puff/neopuff, reported in 30% of low-complexity hospitals, the remaining was reported in more than 80% of the hospitals regardless of the degree of complexity.

**Table 4:** Distribution of hospitals by obstetric and neonatal emergency equipment according to level of complexity, Rio de Janeiro, 2021–2023.

		ICU and NICU		p-value^a^
		No (%)	Yes (%)	
Emergency equipment				
Obstetrics				
	Breathing machine/mechanical ventilator	93.3	100	0.178
	Laryngoscope	100	100	
	Tracheal tube	100	100	
	AMBU	100	100	
	Emergency resuscitation trolley	93.3	100	0.178
	Defibrillator	100	100	
	Cardiac monitor	100	80.8	0.00
	Pulse oximeter	100	100	
	Intrauterine balloon for postpartum hemorrhage	0.0	46.2	0.00
	Non-pneumatic anti-shock garment	6.7	11.1	0.48
	Anesthesia trolley	100	100	
Neonatology				
	Resuscitation unit/table/cradle with radiant heat source	93.3	100	0.17
	Ventilation masks	100	100	
	Pediatric stethoscope	100	100	
	Full laryngoscope	100	100	
	Neonatal endotracheal tube	100	80.8	0.00
	Neonatal tracheal probes without valve	100	100	
	Gastric lavage probe	100	100	
	Meconium aspirator	100	57.7	0.00
	Ventilation material (AMBU or manual resuscitator with oxygen tank)	100	100	
	Aspirator with pressure gauge and oxygen	100	100	
	Baby puff/neopuff	33.3	76.9	0.00
	Pulse oximeter	100	100	
	Pediatric balance	100	100	
	Peripherally inserted central catheter	33.3	81.5	0.00
Drug classes				
	Dopamine agonists	26.7	100	0.00
	Analgesic and anti-inflammatory	100	100	
	Anesthetic	100	100	
	Antibiotic	48.3	100	0.00
	Anticoagulant and antithrombotic	100	100	
	Anticonvulsant	100	100	
	Antiemetics	88.3	100	0.06
	Anti-hemorrhagic	82.9	95.2	0.00
	Antihypertensive	93.3	92.3	0.04
	Antiseptic and keratolytic	68.3	38.5	0.01
	Benzodiazepine	100	100	
	Beta blocker	96.7	100	0.34
	Corticosteroids	100	100	
	Diuretic	100	100	
	Hypoglycemic	100	100	
	Rho(d) immune globulin	100	100	
	Uterine contractility inhibitors	86.7	80.8	0.48
	Surfactant	73.3	100	0.04
	Uterotonic and oxytocic	73.3	88.5	0.04
Support services				
	Imaging tests	73.3	100	0.00
	Laboratory of pathology and clinical analysis	96.7	100	0.34
	Ambulance for parturients’ transportation	100	100	
	Ambulance for NB transportation	100	100	
	Blood bank or transfusion unit	100	100	

ICU: adult intensive care unit; NICU: neonatal intensive care unit; NB: Newborn; AMBU: artificial manual breathing unit.

a p-value of the χ^2^ test.

Some classes of essential drugs were not mentioned in all low-complex maternity hospitals, such as dopamine agonists, antiemetics, antiseptic and keratolytic drugs, beta blockers, surfactants, and uterine contractility inhibitors, the latter also absent in high-complexity maternity hospitals. Other classes did not have one or two essential medicines, including antibiotics, antihypertensive, anti-hemorrhagic, uterotonic, and oxytocic drugs.

The evaluated support services were available 24/7 for almost all maternity hospitals, except for imaging tests, available in 73.3% of the low-complexity maternity hospitals.

In Table 5, we show that in RJ 30.5% of women at high obstetric risk were seen in maternity hospitals without ICU/NICU, with this value consisting of 31.8% in public hospitals and 41.2% in the metropolitan region. In small cities, the percentage decreases to 12.4%. The hospitalization of women at normal risk in maternity hospitals with ICU/NICU was 40.5% in RJ, reaching almost half in mixed hospitals and 70% in small cities of the state.

**Table 5:** Number and proportion of low-risk and high-risk women seen according to level of complexity, geographic location, and hospital type, Rio de Janeiro, 2021–2023.

	ICU/NICU	Obstetric risk		Total (n)	Obstetric risk		Total (%)	p-value^a^
		Low (n)	High (n)		Low (%)	High (%)		
State	No	480	81	561	59.5	30.5	52.3	0.00
	Yes	327	185	512	40.5	69.5	47.7	
Metropolitan Region	No	416	70	486	69.6	41.2	63.3	0.00
	Yes	182	100	282	30.4	58.8	36.7	
Small cities	No	64	12	76	30.8	12.4	24.9	0.00
	Yes	144	85	229	69.2	87.6	75.1	
Public	No	421	67	488	60.7	31.8	53.9	0.00
	Yes	273	144	417	39.3	68.2	46.1	
Mixed	No	59	14	73	52.2	25.5	43.5	0.00
	Yes	54	41	95	47.8	74.5	56.5	

ICU: adult intensive care unit; NICU: neonatal intensive care unit.

a p-value of the χ^2^ test.

## DISCUSSION

According to our results, there is a prevalence of 24.8% of high obstetric risk in RJ. We identified failures in prenatal care, in the integration between prenatal care and delivery care, and in the structure of maternity hospitals, especially in those of low complexity. One-third of high-risk women were assisted in maternity hospitals without ICU/NICU, while 40.5% of women at normal risk were assisted in high-complexity services, demonstrating failures in the hierarchization of obstetric care.

The prevalence of women at high obstetric risk is similar to that found in public hospitals located in the capitals of the states of the South and Southeast regions^8^, and it was higher among women seen in hospitals located in small cities of the state. The highest occurrence was observed in women aged 35 years or over, multiparous and obese. These are characteristics that predispose to the occurrence of the clinical and obstetric conditions evaluated as well as to the development of complications during pregnancy^14^. Low level of education among women at high obstetric risk, which is associated with difficulties in access to healthcare services, makes them vulnerable to unfavorable outcomes. Low adequacy of prenatal care in women seen in public-funded services, which were also the most socially vulnerable, was observed in another study of the NBII research in RJ^15^. Hospitalization during pregnancy, an important marker of maternal morbidity, was almost three times higher among women at high obstetric risk.

Although quality prenatal care, timely and at the appropriate level of complexity, is recognized as important to reduce the chances of obstetric complications and maternal death^16^, our results point to deficiencies in the prenatal services offered, regardless of obstetric risk. The adequacy of this care remains low. Even if there is no marker of severity of the clinical and/or obstetric conditions identified in prenatal care, the expressive percentage of women at high obstetric risk having their prenatal appointments exclusively in primary health care suggests limited access to healthcare services that fully guarantee the specificity of care required by women at obstetric risk.

Among the several factors that may have contributed to the challenges in the effectiveness of managing hypertension, diabetes, and nutritional evaluation in prenatal care, we highlight the incomplete implementation of processes and procedures recommended by technical standards of the Ministry of Health, including the absence of records of procedures in prenatal cards for a considerable percentage of women. These are situations that interfere not only in the identification of complications in pregnancy, but also in the timely intervention, in the difficulty of communication among healthcare professionals, as well as in the chances of having women at high obstetric risk not diagnosed among those without the registration of essential evaluations.

Although it is known that the link between maternity hospital and labor care promotes better obstetric results, especially for pregnant women classified as at high obstetric risk, according to our results, one in five pregnant women has traveled to be hospitalized for delivery care. We highlight the main reason mentioned by women at high obstetric risk for traveling, which was the difficulty in finding a health facility appropriate to their needs, contributing to the delay in receiving appropriate care and to the increase in the risk of severe maternal morbidity and even death.

Corroborating previous studies^9,17^, hospitals with ICU/NICU presented better levels of adequacy in all structure dimensions evaluated. The absence of formal reference service for the delivery of pregnant women and newborns at risk via regulation center in a considerable percentage of maternity hospitals certainly contributes to limiting access to those who most need care in hospitals of high complexity.

Although researchers show that an effective intervention during labor and delivery care depends on the number of qualified professionals available^18,19^, the limited analysis of the profile of the team of healthcare professionals indicates possible gaps in labor and delivery care. The absence of obstetricians, anesthesiologists, and obstetric nurses on a 24-hour on-call shift in low-complexity maternity hospitals^20^ indicates possible failures in the admission examination of women to maternity hospitals, in the follow-up after going into labor and, consequently, a large time interval between the decision to make a cesarean section and its effectiveness. The adverse consequences of the delay, as well as the absence of adequate care for women's needs at the time of delivery, have already been documented by several authors^21,22^.

The most concerning fact is the lack of a team of neonatology professionals on a 24-hour on-call shift in low-complexity facilities, a fact that may hinder the detection of risk conditions of the newborn at birth as well as deficiencies in the care at rooming-in. Medical and nursing coordinators specialists in obstetrics and neonatology services provide greater technical competence for decision-making to carry out an appropriate procedure. The absence of coordinators in obstetric services in low-complexity hospitals is noteworthy, a situation that worsens in neonatology services regardless of the complexity of the health facility. Women at high obstetric risk should be seen in hospitals with specialists to collaborate with the team of maternal and fetal care in hospitals of high complexity, but in this study, we identified low presence of these specialists. Teaching activities in hospitals are more frequent in high-complexity facilities, denoting a more experienced professional team and, therefore, with a greater possibility of a positive effect on the quality of care for women and newborns^23^.

Although recommended by the World Health Organization^24^ for the treatment of obstetric hemorrhage, one of the main causes of maternal death^25^, the low availability of NASG and intrauterine balloon in maternity hospitals is worrying, a fact that may contribute to the maintenance of high levels of maternal mortality. Among the minimum equipment required for newborn's care, it is noteworthy that a significant portion of the hospitals without ICU/NICU reported the unavailability of the baby puff/neopuff, useful in the care of the newborn that requires neonatal resuscitation.

A significant percentage of low-complexity maternity hospitals are not in accordance with the legislation regarding the availability of all drug classes and, in spite of that, they have limited access to certain essential medicines for properly managing obstetric hypertensive emergencies and hemorrhages.

It is known that the access of women at high obstetric risk to an adequate maternity hospital to meet their health needs benefits the health of both women and newborns^26^; therefore, it is worth noting that a significant number of women at high obstetric risk is admitted to hospitals without ICU/NICU^20^. This scenario is aggravated in the metropolitan region, where almost two-thirds of deliveries occurred in maternity hospitals without ICU/NICU, indicating insufficiency in the processes of defining adequate care paths in the face of risk stratification that should result in the link of the pregnant woman to a maternity compatible with gestational and neonatal risk. Researchers of the "Birth in Brazil" survey already pointed to limited access to health facilities of high complexity throughout the country^8^. Furthermore, according to the research data, there is the possibility of greater exposure of women at normal risk to unnecessary interventions^27^, considering that an expressive percentage of them was hospitalized in maternity hospitals with ICU/NICU.

Although there is uncertainty regarding the degree of reliability of hospital structure data, due to the lack of direct observation of the evaluated dimensions, a fact that can introduce biases, in the case of the manager informing a more adequate structure, the choice of the method ensured the participation of all hospitals in the sample and a low percentage of nonresponse. It should be noted that the evaluation limited to the "maternity structure" dimension does not guarantee that the health needs of women who sought care in these facilities were met.

The results give visibility to partial prenatal care and unlinked to labor care, in which a significant number of mothers and newborns were exposed to unnecessary and preventable risks. The incompleteness of the procedures deemed effective for reducing favorable outcomes during prenatal care and gaps in the articulation of the network of available obstetric services, such as traveling in search of hospital vacancies and delivery in inappropriate maternity hospitals, were highlighted. This is true both for women at high obstetric risk, as failures of the hospital structure can interfere in the appropriate care in a timely manner, and for those at normal risk — who, in maternity hospitals with ICU/NICU, may often have their needs for the physiological evolution of labor not met^28^.

The challenges remain, and many efforts are needed to improve labor and delivery care, as recommended by Rede Cegonha^7^. Among the initiatives that must be intensified, we emphasize the need to improve prenatal care and the implementation of an articulated network of services that integrate the SUS, focused on maternity hospitals that guarantee the conditions of structure and work processes for quality and safety in obstetric and neonatal care, both in good practices at normal risk and in the management of complications and at high obstetric risk, in such a way to impact the persistent and considerable outcomes of maternal and neonatal mortality and morbidity.

## Data Availability

The datasets generated and/or analyzed during the present study are not publicly available due to [ethical/legal/privacy] restrictions, but are available from the corresponding author upon request.
