# Supplementary material for: Use of prenatal care and hospital structure according to obstetric risk in Rio de Janeiro, Brazil
Source: Rev Saude Publica. 2025 Oct 20;59(Suppl 1):e3s. doi: 10.11606/s1518-8787.2025059006571 (PMC12539633; doi:10.11606/s1518-8787.2025059006571)
Supplement: Translation

# Translation: Utilização do pré-natal e estrutura hospitalar segundo o risco obstétrico no Rio de Janeiro

**Authors:** Sonia Duarte de Azevedo Bittencourt, Rosa Maria Soares Madeira Domingues, Danielle Portella Ferreira, Ana Paula Esteves-Pereira, Marcos Augusto Bastos Dias, Marcos Nakamura-Pereira, Alessandra do Nascimento Bernardo, Paulo Cesar da Graça Souza Suppo Blengini, Maria Auxiliadora de Souza Mendes Gomes, Maria do Carmo Leal

**Affiliations:** I Fundação Oswaldo Cruz. Escola Nacional de Saúde Pública Sérgio Arouca. Rio de Janeiro, RJ, Brasil; II Fundação Oswaldo Cruz. Instituto Nacional de Infectologia Evandro Chagas. Rio de Janeiro, RJ, Brasil; III Fundação Oswaldo Cruz. Instituto Nacional de Saúde da Mulher, Criança e Adolescente Fernandes Figueira. Rio de Janeiro, RJ, Brasil

**Keywords:** Cuidado Pré-natal, Estrutura dos Serviços, Qualidade da Assistência à Saúde

## Abstract

**OBJETIVO:**

Estimar a utilização e adequação de serviços de pré-natal e verificar a estrutura da maternidade para atender às necessidades de cuidado de saúde durante o parto no Sistema Único de Saúde.

**MÉTODOS:**

Estudo seccional de base hospitalar realizado no estado do Rio de Janeiro. Foram elegíveis 1.073 puérperas cujo parto ocorreu em hospitais públicos e mistos. As entrevistas foram realizadas no hospital e foram extraídos dados da caderneta de pré-natal e do prontuário materno. Para avaliar a estrutura hospitalar foram entrevistados os gestores. As características sociodemográficas, obstétricas e do cuidado pré-natal das puérperas foram descritas segundo o risco obstétrico. A avaliação da estrutura da maternidade englobou: recursos humanos, medicamentos, equipamentos de emergência e serviços de apoio, segundo o nível de complexidade. Ao final, analisou-se a distribuição das puérperas classificadas em risco obstétrico segundo a complexidade da estrutura hospitalar.

**RESULTADOS:**

As maiores prevalências de alto risco obstétrico foram observadas nos hospitais localizados no interior, entre as mulheres brancas, com 35 ou mais de idade, com até 11 anos de estudo, multíparas e obesas. A adequação do pré-natal, o controle da hipertensão arterial, diabetes e da avaliação nutricional foram baixos e a peregrinação foi expressiva. Os hospitais com UTI/UTIN apresentaram melhores níveis de adequação em todas as dimensões de estrutura avaliadas. Ao nível estadual, 30,5% das mulheres de alto risco obstétrico foram atendidas em maternidades sem UTI/UTIN e 40,4% de risco habitual em maternidades com UTI/UTIN.

**CONCLUSÃO:**

O estudo sublinha a necessidade de melhorar a assistência pré-natal e a implementação de uma rede articulada dos serviços que integram o Sistema Único de Saúde (SUS), centrada em maternidades que garantam as condições de estrutura para a qualidade e segurança no cuidado obstétrico e neonatal, tanto nas boas práticas no risco habitual quanto no manejo de intercorrências e no alto risco obstétrico, de forma a impactar os persistentes resultados elevados de morbimortalidade materna e neonatal.

## INTRODUÇÃO

No Brasil, embora a atenção pré-natal e ao parto tenha alcançado cobertura praticamente universal, com quase a totalidade dos partos ocorrendo em hospitais e assistidos por pessoal qualificado^1,2^, a persistência de elevados níveis de morbimortalidade materna por causas evitáveis^3,4^ indica problemas associados aos processos de trabalho durante o cuidado obstétrico e à gestão dos serviços das redes de atenção para a saúde sexual e reprodutiva^5,6^. São situações que contribuem para ampliar as iniquidades da gestante em obter serviços de saúde adequados às suas necessidades, assim como nos resultados da assistência recebida.

Com o propósito de transformar este cenário, o Ministério da Saúde implementou, em 2011, a estratégia Rede Cegonha^7^, que tem como base o desenvolvimento da linha de cuidado à gestante com base na classificação das necessidades de saúde de cada mulher, de modo a estruturar a articulação das responsabilidades e funções de cada serviço de saúde envolvido na assistência ao pré-natal e ao parto, e assim garantir o cuidado integral, oportuno e resolutivo.

No entanto, mais de uma década após a implantação da Rede Cegonha, mesmo quando são identificados riscos durante o período pré-natal, é difícil garantir a internação em maternidades com estrutura compatível, tanto com a necessidade de cuidado da gestante como com a do recém-nascido^8,9^.

Essa situação torna-se mais dramática na emergência obstétrica, que demanda agilidade dos mecanismos de referência. A dificuldade de comunicação entre os serviços e as centrais de regulação responsáveis por viabilizar a transferência para serviços com estrutura compatível tem sido apontada como fator associado ao atraso na obtenção do cuidado adequado^3^. A regulação da assistência ao alto risco materno, um dos desafios a superar para a organização da atenção materno-infantil, faz parte das prioridades do Plano Estadual de Saúde (2020–2023)^10^ do estado do Rio de Janeiro (RJ).

Este estudo teve por objetivo estimar a utilização e adequação de serviços de atenção pré-natal e verificar a estrutura da maternidade para atender às necessidades de cuidado de saúde durante o parto no Sistema Único de Saúde (SUS) no RJ.

## MÉTODOS

Trata-se de um estudo transversal de base hospitalar e abrangência nacional ("Nascer no Brasil II" — NBII). Foi selecionada uma amostra probabilística em dois estágios (hospital e puérperas), conforme protocolo do estudo publicado em Leal et al.^11^. Para este estudo foram elegíveis as puérperas de nascido vivo (NV) com qualquer peso ou idade gestacional e puérperas de natimortos com idade gestacional ≥ 22 semanas ou peso ≥ 500 g.

### Estudo no Rio de Janeiro

No RJ a amostra de puérperas foi calculada com base na proporção de cesarianas (57%) em 2019, nível de significância de 5% e potência de 90% para detectar diferenças de 7%. Utilizou-se um efeito de desenho de 1,3, resultando no tamanho amostral mínimo de 1.350 puérperas. Para alcançar esse número, a amostra de puérperas do RJ foi de 30 nos estabelecimentos com 100–499 NV/ano e de 90 nos hospitais com ≥ 500 NV/ano. Cálculos *post hoc* indicam que a amostra tem poder de detectar diferenças absolutas de 5% para desfechos com prevalência entre 5 e 20%.

Foram incluídos nesta análise os 17 hospitais classificados como públicos (financiamento e gestão pelo setor público) e mistos (financiamento público e privado e gestão privada), representando 86 hospitais do RJ. Nos hospitais públicos, foram incluídas todas as puérperas entrevistadas, enquanto nos hospitais mistos foram incluídas apenas aquelas que tiveram parto com financiamento público, correspondendo a 86,5% das entrevistadas nesses hospitais.

A coleta de dados, realizada de 2021 a 2023, incluiu entrevista com as mulheres no puerpério imediato, extração de dados de cadernetas de pré-natal e de dados clínicos dos prontuários maternos após a alta hospitalar. Puérperas que não apresentavam dados de cadernetas de pré-natal foram excluídas da análise. As informações sobre estrutura da maternidade foram obtidas por meio de entrevista com o gestor de cada unidade hospitalar. Informações detalhadas sobre os instrumentos de coleta são relatadas em Leal et al.^11^.

Para estimar as necessidades de cuidado de saúde, em cada uma das maternidades do estudo as puérperas foram classificadas segundo o risco obstétrico^8^, sendo consideradas de alto risco aquelas que apresentaram pelo menos uma das seguintes condições clínicas e/ou obstétricas identificadas no pré-natal: hipertensão crônica ou gestacional, diabetes pré-gestacional ou gestacional, doença renal crônica, doença cardíaca, lúpus, esclerodermia, infecção pelo HIV e outras infecções no momento da admissão hospitalar e gestação gemelar. As demais puérperas foram classificadas como de risco habitual. Foram analisadas as seguintes variáveis por risco obstétrico: localização do hospital (região metropolitana e interior), tipo do hospital (público e misto), idade (10–19, 20–34 e 35 ou mais), cor da pele (branca e negra), anos de estudo (até 11, 12 anos ou mais), paridade (primíparas e multíparas) e índice de massa corporal pré-gestacional (IMC < 25, IMC ≥ 25).

Foi verificada a assistência pré-natal segundo o risco obstétrico por meio dos seguintes indicadores: adequação do pré-natal (início até 12ª semana de gestação e número de consultas segundo a idade gestacional conforme o calendário recomendado pela Organização Mundial da Saúde^12^, tipo de unidade de saúde onde a mulher realizou as consultas de pré-natal; registro de peso corporal da mulher em três ocasiões, da altura, razão da medição de pressão arterial por consulta e da realização de exames de glicemia. A integração da assistência pré-natal ao parto foi analisada pela proporção de mulheres orientadas sobre o local da internação para o parto, a proporção de mulheres que buscaram mais de um serviço no momento para internação para o parto e os motivos da peregrinação.

Para estimar o nível de complexidade da assistência ao parto, os hospitais da amostra foram classificados segundo a existência de unidade de terapia intensiva de adulto (UTI) e unidade de terapia intensiva neonatal (UTIN). Para as maternidades de alta complexidade foi perguntado se era referência formal pelo sistema de regulação para parto de gestante de risco. Para averiguar a dimensão "recursos humanos", foi investigada a disponibilidade de pelo menos um de cada profissional (obstetra, neonatologista, anestesista, enfermeiro obstetra e neonatologista) de plantão durante 24 horas; a existência de coordenador médico e de enfermagem com título de especialista nos serviços de obstetrícia e neonatologia; a disponibilidade de especialistas de retaguarda para suporte no caso de mulheres com complicações; e se o hospital era campo de prática de ensino de residência médica e/ou de enfermagem em obstetrícia e/ou neonatologia. Nas dimensões "medicamentos" e "equipamentos", foi verificada a disponibilidade de 20 classes de medicamentos^13^ e de um conjunto de equipamentos para o atendimento emergencial à gestante e ao recém-nascido^13^. Com relação à dimensão "serviços de apoio" foi verificado o acesso a banco de sangue ou unidade transfusional, laboratório de patologia e análises clínicas, exame de imagem e de ambulância para transporte da mulher e do recém-nascido.

Em seguida, analisou-se a distribuição das puérperas classificadas em risco obstétrico segundo a complexidade da estrutura hospitalar por tipo de hospital e localização. Para a comparação entre as categorias, foi usado o teste do χ^2^, sendo considerado significativo um valor de p < 0,05.

Todas as análises foram realizadas com o *software* Statistical Package for the Social Sciences (SPSS) versão 21 (SPSS Inc., Chicago, Estados Unidos), com inclusão do efeito de desenho, ponderação e calibração. Para a amostra do RJ, foram utilizados para calibração grupos compostos da combinação de estrato, tipo de parto (vaginal, cesariana) e idade da mulher (10–19, 20–34, ≥ 35), utilizando o SINASC-2022 como referência.

A pesquisa NBII foi aprovada pela Comissão Nacional de Ética em Pesquisa, Certificado de Apresentação para Apreciação Ética (CAAE): 21633519.5.0000.5240, em 11 de março de 2020, e teve a aprovação dos comitês locais de ética em pesquisa das instituições ou da diretoria clínica quando da ausência dos comitês locais.

## RESULTADOS

Foram analisadas 1.073 puérperas, correspondendo a 82,2% da amostra das puérperas entrevistadas nas maternidades com financiamento público. Do total de mulheres analisadas, 266 (24,8%) foram classificadas como alto risco obstétrico. Maior prevalência de alto risco obstétrico foi observada nos hospitais localizados no interior do estado, entre as mulheres brancas, com 35 ou mais anos de idade, com até 11 anos de estudo, nas multíparas e nas obesas. Antes da internação para o parto, 18,6% das mulheres com alto risco obstétrico declararam ter sido internadas durante a gravidez, percentual quase três vezes maior que o encontrado entre as mulheres de risco habitual (Tabela 1).

**Tabela 1:** Número e percentual de mulheres com alto risco obstétrico segundo características do hospital e maternas. Estado do Rio de Janeiro, 2021–2023.

		Risco obstétrico		Total n	%	Valor p^a^
		Baixo	Alto			
Total		807	266	1.073	24,8	
Localização						
	Região Metropolitana	599	169	768	22,0	0,00
	Interior	208	97	305	31,8	
Tipo de hospital						
	Público	694	211	905	23,3	0,00
	Misto	113	55	168	32,7	
Grupo de idade (anos)						
	10 a 19	119	27	146	18,5	0,00
	20 a 34	605	188	793	23,7	
	35 ou mais	84	51	135	37,8	
Cor da pele						
	Branca	178	78	256	30,5	0,05
	Negra (preta, parda e indígena)	629	188	817	23,0	
Anos de estudo						
	Até 11	422	163	585	27,9	0,01
	≥ 12	385	103	488	21,1	
Paridade						
	Primípara	350	94	444	21,2	0,01
	Multípara	457	173	630	27,5	
Índice de Massa Corporal						
	Até 24	528	138	666	20,7	0,00
	≥ 25	154	88	242	36,4	
Gestante internada alguma vez durante esta gravidez?						
	Não	744	214	958	6,7	0,00
	Sim	54	49	103	18,6	

a Valor de p de teste χ^2^.

Como pode ser observado na Tabela 2, a adequação do pré-natal, considerando-se época de início e número de consultas, foi de 71,5%, sendo um pouco mais de 90% das gestantes assistidas em uma única unidade, preferencialmente na atenção básica. A aferição da pressão estava registrada em 88,1% das consultas, primeira glicemia em 78,1% e segunda glicemia em 30%. Apenas um terço das gestantes apresentava registro completo de pressão arterial e exames de glicemia. O registro de peso estava anotado em três momentos da assistência ao pré-natal em 90% das cadernetas de pré-natal, mas o registro da altura foi reduzido, limitando a avaliação nutricional (40% em mulheres de alto risco obstétrico). Mulheres de alto risco obstétrico apresentaram maior registro do segundo exame de glicemia, sem outras diferenças em relação às de risco habitual. Apenas 16,8% foram assistidas em unidades ambulatoriais de referência.

**Tabela 2:** Número e distribuição de indicadores da assistência pré-natal de puérperas segundo risco obstétrico, Rio de Janeiro, 2021–2023.

		Risco obstétrico		Total n	Risco obstétrico		Total %	Valor p^a^
		Baixo n	Alto n		Baixo %	Alto %		
Adequação do número de consultas segundo idade gestacional no parto								
	Sim	566	194	760	70,8	73,4	71,5	0,40
	Não	233	70	303	29,2	26,5	28,5	-
Organização do pré-natal								
Assistência pré-natal realizada apenas em um tipo de unidade de saúde								
	Sim	747	239	986	92,5	89,8	91,8	0,33
	Não	60	27	87	7,5	10,2	8,2	-
Tipo de unidade de saúde onde a mulher realizou as consultas de pré-natal								
	Atenção básica de saúde	623	179	802	83,5	74,9	81,4	0,00
	Ambulatório (público, privado e plano)	65	40	105	8,7	16,7	10,7	-
	Outros (consultório grupo, casa de partos)	58	20	78	7,8	8,4	7,9	-
Procedimentos								
	Pesagem na 1ª consulta, 24ª a 28ª semana e última consulta	651	219	870	85,4	88,7	86,2	0,53
	Aferição de altura	405	110	515	50,2	41,4	48,0	0,01
	Todas as aferições de peso e altura	355	100	455	44,0	37,6	42,4	0,06
	Menos de uma medição de pressão arterial por consulta	668	221	889	87,8	89,1	88,1	0,57
	Exame da 1ª glicemia	634	204	838	78,6	76,7	78,1	0,52
	Exame da 2ª glicemia	255	112	367	31,6	42,1	34,2	0,02
	Pressão arterial (pelo menos uma por consulta) e exames de glicemia	229	96	325	28,4	36,1	30,3	0,01
	Orientada a visitar a maternidade	121	30	151	15,0	11,3	14,1	0,12
	Peregrinação para o parto	121	48	169	15,0	18,0	15,8	0,08
Motivos da peregrinação								
	Não havia vaga	21	8	29	17,8	17,8	17,8	0,72
	Não estava em trabalho de parto	37	14	51	31,4	31,1	31,3	0,65
	Referenciada para outra maternidade por situação de risco	18	14	32	15,3	31,1	19,6	0,01
	Referenciada para outra maternidade, mas não era situação de risco	18	2	20	15,3	4,4	12,3	0,12
	Maternidade sem médico ou sem condição de atender	6	5	11	5,1	11,1	6,7	0,11
	Outro	18	2	20	15,3	4,4	12,3	0,12

a Valor de p de teste χ^2^.

Ainda na Tabela 2, somente 14,1% das gestantes foram orientadas a visitar a maternidade para conhecer o ambiente em que ficariam durante o trabalho de parto e parto, ficando a diferença por risco obstétrico não significativa. Verifica-se que a peregrinação das mulheres no momento da internação para o parto foi mais frequente entre as mulheres de alto risco obstétrico, embora também sem significância estatística. As principais razões citadas foram semelhantes entre os dois grupos: não havia vaga e a mulher não estava em trabalho de parto, com exceção dos casos em que o motivo de ser referenciada para outra maternidade era situação de risco, declarada por mais de um terço das mulheres de alto risco obstétrico.

A Tabela 3 mostra que 30,2% dos hospitais da amostra dispunham de UTI/UTIN, sendo 34,3% entre os públicos e 15,8 % dos mistos, sem diferença na distribuição entre a região metropolitana e o interior. Dessas maternidades, 46,1% informaram ser referência formal pelo sistema de regulação para internação e parto de gestante e recém-nascidos de risco (dados não apresentados nas tabelas).

**Tabela 3:** Distribuição dos hospitais por localização, tipo de financiamento e disponibilidade de recursos humanos segundo nível de complexidade, Rio de Janeiro, 2021–2023.

		UTI e UTIN				Valor p^a^
		Não		Sim		
		n	%	n	%	
Tipo de hospital						
	Público	44	65,7	23	34,3	0,12
	Misto	16	84,2	3	15,8	-
Localização						
	Região Metropolitana	40	71,4	16	28,6	0,64
	Interior	20	66,7	10	33,3	-
Recursos humanos Obstetrícia (em plantão de 24 horas)						
	Obstetra	60	100	26	100	-
	Anestesista	48	80,0	26	100	0,01
	Enfermeira obstétrica	40	66,7	19	73,1	0,35
	Todos	16	26,7	19	73,1	0,00
	Coordenador médico especialista em obstetrícia	60	100	26	100	-
	Coordenador enfermagem especialista em obstetrícia	17	28,3	21	80,8	0,00
Neonatologia (em plantão de 24 horas)						
	Neonatologista	2	3,3	26	100	0,00
	Anestesista	3	5,0	5	19,2	0,03
	Enfermeira neonatal	12	20,0	19	70,4	0,00
	Todos	0	0	5	18,5	
	Coordenador médico especialista em neonatologia	18	69,2	26	100	0,00
	Coordenador enfermagem especialista em neonatologia	6	23,1	26	100	0,00
Presença de especialista^b^						
	Nenhum	24	40,7	0	0	0,00
	Todos	3	5,1	10	37,0	0,00
Oferta de residência médica e/ou de enfermagem em obstetrícia ou neonatologia		4	6,7	17	63,0	0,00

UTI: unidade de terapia intensiva de adulto; UTIN: unidade de terapia intensiva neonatal.

a Valor de p de teste χ^2^.

b Especialidade médica: Cardiologia, Neurologia; Nefrologia; Hematologia; Infectologia e Cirurgião Geral.

Menores proporções de recursos humanos disponíveis em plantão de 24 horas foram relatadas nos hospitais de menor complexidade, principalmente neonatologistas (3,3%), anestesistas (5%) e enfermeiras (20%) no serviço de neonatologia. A disponibilidade de anestesista foi pouco frequente mesmo em serviços de alto risco (19,2%), diferindo das demais categorias profissionais, em que essa disponibilidade foi superior a 70%. Os hospitais de menor complexidade apresentam a menor frequência de coordenadores médicos e enfermagem nos serviços de obstetrícia e neonatologia, e de médicos especialistas de retaguarda para os casos de mulheres com complicação (40,7%). Apenas 6,7% dos hospitais oferecem curso de residência médica e/ou enfermagem (Tabela 3).

A Tabela 4 mostra a disponibilidade de equipamentos essenciais e estratégicos para o cuidado materno e neonatal em emergências. Para a atenção à mulher, o traje antichoque não pneumático (TAN) foi o equipamento menos frequente para todas as maternidades; já o balão intrauterino não foi declarado em nenhuma maternidade de menor complexidade e em menos da metade entre aquelas de maior complexidade. Para a atenção ao recém-nascido, com exceção da baixa disponibilidade de *baby puff/ neo puff*, declarada em 30% dos hospitais de menor complexidade, o restante foi declarado em mais de 80% dos hospitais independentemente do grau de complexidade.

**Tabela 4:** Distribuição dos hospitais por equipamentos de emergência obstétrica e neonatal segundo nível de complexidade, Rio de Janeiro, 2021–2023.

		UTI e UTIN		Valor p^a^
		Não (%)	Sim (%)	
Equipamentos de emergência				
Obstetrícia				
	Respirador/ventilador mecânico	93,3	100	0,178
	Laringoscópio	100	100	
	Tubo orotraqueal	100	100	
	AMBU	100	100	
	Carrinho de parada cardiorrespiratória	93,3	100	0,178
	Desfibrilador	100	100	
	Monitor cardioscópio	100	80,8	0,00
	Oxímetro de pulso	100	100	
	Balão intrauterino para atendimento à hemorragia pós-parto	0,0	46,2	0,00
	Traje antichoque não pneumático	6,7	11,1	0,48
	Carrinho de anestesista	100	100	
Neonatologia				
	Unidade/mesa/berço para reanimação com fonte de calor radiante	93,3	100	0,17
	Máscaras de ventilação	100	100	
	Estetoscópio clínico infantil	100	100	
	Laringoscópio completo	100	100	
	Tubo endotraqueal neonatal	100	80,8	0,00
	Sondas traqueais neonatais sem válvula	100	100	
	Sondas de aspiração gástrica	100	100	
	Dispositivo para aspiração de mecônio	100	57,7	0,00
	Material para ventilação (AMBU ou ressuscitador manual com reservatório de oxigênio)	100	100	
	Aspirador com manômetro e oxigênio	100	100	
	*Baby puff/ neo puff*	33,3	76,9	0,00
	Saturímetros	100	100	
	Balança pediátrica	100	100	
	Cateter central de inserção periférica	33,3	81,5	0,00
Classes de medicamentos				
	Agonista dopaminérgico	26,7	100	0,00
	Analgésico e Anti-inflamatório	100	100	
	Anestésico	100	100	
	Antibiótico	48,3	100	0,00
	Anticoagulante e Antitrombótico	100	100	
	Anticonvulsivo	100	100	
	Antieméticos	88,3	100	0,06
	Anti-hemorrágico	82,9	95,2	0,00
	Anti-hipertensivo	93,3	92,3	0,04
	Antisséptico e ceratolítico	68,3	38,5	0,01
	Benzodiazepínico	100	100	
	Betabloqueador	96,7	100	0,34
	Corticosteroides	100	100	
	Diurético	100	100	
	Hipoglicemiante	100	100	
	Imunoglobulina RH(D)	100	100	
	Inibidor da contratilidade uterina	86,7	80,8	0,48
	Surfactante	73,3	100	0,04
	Uterotônico e ocitócito	73,3	88,5	0,04
Serviços de apoio				
	Exames de imagem	73,3	100	0,00
	Laboratório de patologia e análises clínicas	96,7	100	0,34
	Ambulância para transporte de parturiente	100	100	
	Ambulância para transporte de RN	100	100	
	Banco de sangue ou unidade transfusional	100	100	

UTI: unidade de terapia intensiva de adulto; UTIN: unidade de terapia intensiva neonatal; RN: recém-nascido; AMBU: artificial manual breathing unit (unidade de ventilação artificial manual)

a Valor de p de teste χ^2^.

Algumas classes de medicamentos essenciais não foram mencionadas em todas as maternidades de baixa complexidade, sendo elas: agonistas dopaminérgicos, antieméticos, antissépticos e ceratolíticos, betabloqueador, surfactantes e inibidores da contratilidade uterina, estes últimos também ausentes em maternidades de alta complexidade. Outras classes não tinham um ou dois medicamentos essenciais, entre eles os antibióticos, anti-hipertensivos, anti-hemorrágicos, uterotônicos e ocitócitos.

Os serviços de apoio avaliados estavam disponíveis 24 horas por dia durante sete dias da semana para a quase totalidade das maternidades, com exceção do exame de imagem, disponível em 73,3% das maternidades de menor complexidade.

Na Tabela 5, observa-se que no RJ 30,5% das mulheres de alto risco obstétrico foram atendidas em maternidades sem UTI/UTIN, sendo esse valor de 31,8% nos hospitais públicos e 41,2% na região metropolitana. Já no interior o percentual se reduz para 12,4 %. A internação de mulheres de risco habitual em maternidades com UTI/UTIN foi de 40,5% no RJ, alcançando quase a metade nos hospitais mistos e 70% no interior do estado.

**Tabela 5:** Número e proporção de mulheres de baixo risco e alto risco atendidas segundo nível de complexidade, localização geográfica e tipo de hospital, Rio de Janeiro, 2021–2023.

	UTI/UTIN	Risco obstétrico		Total (n)	Risco obstétrico		Total (%)	Valor p^a^
		Baixo (n)	Alto (n)		Baixo (%)	Alto (%)		
Estado	Não	480	81	561	59,5	30,5	52,3	0,00
	Sim	327	185	512	40,5	69,5	47,7	
Região Metropolitana	Não	416	70	486	69,6	41,2	63,3	0,00
	Sim	182	100	282	30,4	58,8	36,7	
Interior	Não	64	12	76	30,8	12,4	24,9	0,00
	Sim	144	85	229	69,2	87,6	75,1	
Público	Não	421	67	488	60,7	31,8	53,9	0,00
	Sim	273	144	417	39,3	68,2	46,1	
Misto	Não	59	14	73	52,2	25,5	43,5	0,00
	Sim	54	41	95	47,8	74,5	56,5	

UTI: unidade de terapia intensiva de adulto; UTIN: unidade de terapia intensiva neonatal.

a Valor de p de teste χ^2^.

## DISCUSSÃO

Os resultados deste estudo revelam prevalência de 24,8% de alto risco obstétrico no RJ, sendo identificadas falhas na assistência pré-natal, na integração da assistência pré-natal e ao parto e na estrutura das maternidades, principalmente naquelas de menor complexidade. Um terço das mulheres de alto risco foi assistida em maternidades sem UTI/UTIN, enquanto 40,5% das mulheres de risco habitual foram assistidas nesses serviços de maior complexidade, revelando falhas na hierarquização do cuidado obstétrico.

A prevalência de mulheres de alto risco obstétrico se aproxima do encontrado nos hospitais públicos localizados nas capitais dos estados das Regiões Sul e Sudeste^8^, tendo sido maior entre as mulheres atendidas nos hospitais localizados no interior do estado. A maior ocorrência foi observada em mulheres com 35 anos ou mais de idade, multíparas e obesas. São características que predispõem à ocorrência das condições clínicas e obstétricas avaliadas, assim como ao desenvolvimento de complicações na gestação^14^. A menor escolaridade entre mulheres de alto risco obstétrico, condicionante associado às dificuldades de acesso a serviços de saúde, as torna vulneráveis aos desfechos desfavoráveis. Menor adequação da assistência pré-natal em mulheres assistidas em serviços com financiamento público, que também eram as mais vulneráveis socialmente, foi observada em outro estudo do NBII no RJ^15^. A hospitalização durante a gestação, importante marcador de morbidade materna, foi quase três vezes maior entre mulheres de alto risco obstétrico.

Embora a atenção pré-natal de qualidade, em tempo oportuno e no nível de complexidade necessário, seja reconhecida como importante para reduzir as chances de complicações obstétricas e morte materna^16^, os resultados deste estudo mostram deficiências dos serviços de pré-natal ofertados, independentemente do risco obstétrico. A adequação dessa assistência permanece baixa. Mesmo que não se tenha um marcador de gravidade das condições clínicas e/ou obstétricas identificadas no pré-natal, o percentual expressivo de mulheres de alto risco obstétrico realizando suas consultas de pré-natal exclusivamente em unidades da atenção básica sugere o acesso limitado aos serviços de saúde que garantam de forma integral a especificidade do cuidado demandado por mulheres com risco obstétrico.

Entre os diversos fatores que podem ter contribuído para os desafios na efetividade do manejo da hipertensão arterial, diabetes e da avaliação nutricional no pré-natal, destacam-se: a realização incompleta de processos e procedimentos preconizados por normas técnicas do Ministério da Saúde, incluindo a ausência de registros de procedimentos nas cadernetas de pré-natal para um percentual importante de mulheres. São situações que interferem não só na identificação de intercorrências na gravidez quanto na intervenção oportuna, na dificuldade de comunicação entre os profissionais de saúde, assim como na possibilidade de existirem mulheres de alto risco obstétrico não diagnosticadas entre as sem registro de avaliações essenciais.

Embora seja sabido que a vinculação da maternidade para a atenção ao parto promova melhores resultados obstétricos, sobretudo para as gestantes classificadas como de alto risco obstétrico, os resultados deste estudo demonstram que uma em cada cinco gestantes peregrinou para conseguir a internação para assistência ao parto. Destaca-se a principal razão citada pelas mulheres de alto risco obstétrico para a peregrinação, que foi a dificuldade em chegar a uma unidade adequada às suas necessidades, contribuindo para a demora em receber cuidado apropriado e para o aumento no risco de morbidade materna grave e até morte.

Corroborando estudos anteriores^9,17^, os hospitais com UTI/UTIN apresentaram melhores níveis de adequação em todas as dimensões de estrutura avaliadas. A ausência de serviço de referência formal para o parto de gestante e recém-nascido de risco via central de regulação em percentual relevante das maternidades, certamente, contribui para limitar o acesso àqueles que mais necessitam de cuidados aos hospitais de maior complexidade.

Por mais que estudos demonstrem que uma intervenção eficaz durante a assistência ao parto e nascimento depende do número de profissionais qualificados disponíveis^18,19^, a análise restrita do perfil da equipe de profissionais de saúde assinala eventuais lacunas na assistência ao parto e nascimento. A inexistência de obstetra, anestesista e enfermeira obstetra 24 horas ininterruptas, em maternidades de menor complexidade^20^, indica a possibilidade de falhas no exame de admissão da mulher às maternidades, no acompanhamento da evolução do trabalho de parto e, consequentemente, um intervalo de tempo grande entre a decisão de fazer uma cesariana e sua efetivação. As consequências adversas da demora, assim como a ausência de atendimento adequado às necessidades da mulher no momento do parto, já foram documentadas por diversos autores^21,22^.

Mais preocupante é a inexistência de equipe de profissionais de saúde da neonatologia 24 horas ininterruptas entre os estabelecimentos de baixa complexidade, fato que pode dificultar a detecção de condições de risco do recém-nascido ao nascimento, assim como deficiências no atendimento no alojamento conjunto. Coordenadores médicos e enfermeiros especialistas nos serviços de obstetrícia e neonatologia aportam maior competência técnica para a tomada de decisão quanto à realização de um procedimento adequado. Impressiona a ausência de coordenadores nos serviços de obstetrícia nos hospitais de baixa complexidade, situação que se agrava para os serviços de neonatologia independentemente da complexidade do estabelecimento. As mulheres de alto risco obstétrico devem ser atendidas em hospitais que contem com especialistas para colaborar com a equipe de cuidados materno e fetal, em hospitais de alta complexidade, mas neste estudo identificamos baixa presença desses profissionais especializados. Atividades de ensino nos hospitais são mais frequentes naqueles de maior complexidade, denotando uma equipe profissional mais experiente e, portanto, com maior possibilidade de efeito positivo na qualidade da assistência à mulher e ao recém-nascido^23^.

Embora recomendados pela Organização Mundial da Saúde^24^ para o tratamento da hemorragia obstétrica, uma das principais causas de morte materna^25^, é preocupante a baixa disponibilidade de TAN e balão intrauterino nas maternidades, fato que pode contribuir para a manutenção dos elevados níveis de mortalidade materna. Entre os equipamentos mínimos para o atendimento do recém-nascido, chama atenção que parcela significativa dos hospitais sem UTI/UTIN declara não ter disponível *baby puff/ neo puff*, útil na assistência ao recém-nascido que demanda reanimação neonatal.

Um percentual significativo das maternidades de baixa complexidade não se encontra em conformidade com a legislação quanto à disponibilidade de todas as classes de medicamentos e, ainda sim, com acesso limitado a determinados medicamentos essenciais para o manejo adequado das emergências hipertensivas obstétricas e das hemorragias.

É sabido que o acesso às mulheres de alto risco obstétrico a uma maternidade adequada para atender às suas necessidades de saúde traz ganhos à saúde da mulher e do recém-nascido^26^; por isso, é sério constatar que um número significativo de mulheres de alto risco obstétrico é admitido em hospitais sem UTI/UTIN^20^. Esse cenário é agravado na região metropolitana, onde quase dois terços dos partos ocorreram em maternidades sem UTI/UTIN, indicando insuficiência nos processos de definição dos caminhos assistenciais adequados diante da estratificação de risco que deveria resultar na vinculação da gestante a uma maternidade compatível com o risco gestacional e neonatal. O estudo "Nascer no Brasil" já apontava o acesso limitado aos estabelecimentos de saúde de maior complexidade em todo o país^8^. Os dados também apontam a possibilidade de maior exposição de mulheres de risco habitual às intervenções desnecessárias^27^, uma vez que um percentual expressivo delas foi internada em maternidades com UTI/UTIN.

Embora haja incerteza quanto ao grau de confiabilidade dos dados de estrutura hospitalar, pela ausência de observação direta das dimensões avaliadas, fato que pode introduzir vieses, no caso de o gestor informar uma estrutura mais adequada, a escolha do método garantiu a participação de todos os hospitais da amostra e baixo percentual de não resposta. É importante salientar que a avaliação restrita à dimensão da estrutura da maternidade não garante que as necessidades de saúde das mulheres que buscaram assistência nesses estabelecimentos foram atendidas.

Os resultados dão visibilidade a uma assistência parcial do pré-natal e desarticulada da assistência ao parto, em que uma parcela significativa de mães e neonatos foi exposta a riscos desnecessários e evitáveis. Foram destacadas a incompletude dos procedimentos considerados efetivos para a redução de desfechos favoráveis durante a assistência pré-natal e lacunas na articulação da rede de serviços obstétricos disponíveis, como a peregrinação da gestante em busca de vaga hospitalar e o parto em maternidade inadequada. Isso se passa tanto para as mulheres com alto risco obstétrico, já que as falhas da estrutura hospitalar podem interferir no cuidado adequado em tempo oportuno, como para as de risco habitual, que em maternidades com UTI/UTIN podem ter as suas necessidades para a evolução fisiológica do trabalho de parto, muitas vezes, não atendidas^28^.

Os desafios permanecem, e muitos esforços são necessários para melhorar a assistência ao parto e nascimento, conforme preconizado pela Rede Cegonha^7^. Entre as iniciativas que devem ser intensificadas, destacam-se a necessidade de melhorar a assistência pré-natal e a implementação de uma rede articulada dos serviços que integram o SUS, centrada em maternidades que garantam as condições de estrutura e processos de trabalho para a qualidade e segurança no cuidado obstétrico e neonatal, tanto nas boas práticas no risco habitual quanto no manejo de intercorrências e no alto risco obstétrico, de forma a impactar os persistentes e elevados resultados de morbimortalidade materna e neonatal.

## Data Availability

Os conjuntos de dados gerados e/ou analisados durante o presente estudo não estão publicamente disponíveis devido a restrições [éticas/legais/de privacidade], mas estão disponíveis com o autor correspondente mediante solicitação.
